# Psychological and Cognitive Sequelae of COVID‐19: Systematic Review and Meta‐Analysis

**DOI:** 10.1111/jpm.70139

**Published:** 2026-05-08

**Authors:** Eduarda Boufleuer, Tainara Wink Vieira, Victória Tiyoko Moraes Sakamoto, Fernando Anschau, Juliana Petri Tavares, Fábio Fernandes Dantas Filho, Daiane Dal Pai

**Affiliations:** ^1^ Postgraduate Program in Nursing Federal University of Rio Grande do Sul Porto Alegre Brazil; ^2^ State Health Department of Rio Grande do Sul Porto Alegre Brazil; ^3^ Grupo Hospitalar Conceição Porto Alegre Brazil; ^4^ Postgraduate Program in Pulmonary Sciences Federal University of Rio Grande do Sul Porto Alegre Brazil

**Keywords:** cohort studies, COVID‐19, long COVID, mental health, meta‐analysis, post‐acute COVID‐19 syndrome, systematic review

## Abstract

**Introduction:**

COVID‐19 pandemic has exacerbated global mental health problems with the development of persistent symptoms.

**Aim:**

To conduct a prevalence meta‐analysis of persistent psychological and cognitive sequelae of COVID‐19 based on cohort studies.

**Method:**

This study followed PRISMA. Cohort studies that followed individuals for at least 12 weeks post‐COVID‐19 were included and pediatric studies were excluded. The databases Embase, Lilacs/BVS, PubMed, SciELO and Scopus were searched in November 2023. Meta‐analyses were performed with subgroup analyses conducted. Results were presented with forest plot graphs and tables. Risk of bias and methodological quality were assessed using Eggers's Test and the Newcastle‐Ottawa Scale.

**Results:**

2,456 studies were identified and screened. Forty‐seven articles were included in the systematic review, and 46 in the meta‐analysis (192,158 participants). The most prevalent psychological outcome was anxiety (0.17; 95% CI 0.11–0.27, 19 studies), followed by cognitive impairments (0.15; 95% CI 0.11–0.20, 35 studies), sleep disturbances (0.14; 95% CI 0.09–0.19, 33 studies) and depression (0.11; 95% CI 0.06–0.19, 16 studies).

**Discussion:**

The mental health consequences of COVID‐19 highlight the need for long‐term monitoring and represent a significant public health challenge. The limitation is the heterogeneity among the studies.

**Implications for Practice/Recommendations:**

These findings represent a public health issue emphasizing the need for public policies and support strategies to mitigate consequences.

**Conclusion:**

Although high prevalence rates were identified, the prediction intervals were wide and heterogeneity remained high–common characteristics of studies conducted during the pandemic.

**Registration:**

Registered in the international Prospective Register of Ongoing Systematic Reviews (PROSPERO) under number CRD42023460632, on September 5, 2023.

## Accessible Summary

**What Is Known on the Subject?:**

- In addition to the high morbidity and mortality of COVID‐19, which affected millions of people, the World Health Organization defines the ongoing public health issue that persists as long COVID, with impairments that continue to affect the quality of life of individuals who had the disease.

**What the Paper Adds to Existing Knowledge?:**

- This is a large study, with meta‐analysis of multiple outcomes (total of 4 outcomes) and 192,158 participants. The most prevalent outcomes were anxiety (17%) and cognitive impairments (15%). The study is relevant due to the ongoing impacts of the pandemic on health systems.
- This systematic review consolidates current evidence on the prevalence and characteristics of psychological and cognitive impairments following COVID‐19 and highlights the heterogeneity of available studies and the methodological differences.

**What Are the Implications or Impacts of the Paper?:**

- The results provide evidence that the problems resulting from COVID‐19 include long‐lasting impairments, highlighting persistent symptoms of anxiety, followed by cognitive impairments, sleep disturbances and depression, which have implications for mental health nursing practices.

**Rigour:**

- Systematic review with meta‐analysis of cohort studies, including 192,158 participants followed for at least 12 weeks after infection (post‐COVID).
- The protocol was registered on the PROSPERO platform under number CRD42023460632 and followed PRISMA criteria.
- Study selection was conducted by two independent reviewers, with a third reviewer resolving any disagreements. Agreement was assessed using the kappa statistic. Risk of bias and the methodological quality of the studies were evaluated using Egger's Test and the Newcastle‐Ottawa Scale.

**What Should People Living With Long COVID Symptoms Take Away From This?:**

- Persistent symptoms after COVID‐19 can vary over time and gradual improvement may be observed with appropriate follow‐up.
- Post‐infection monitoring with healthcare professionals is important, as the healthcare team can assist in identifying symptoms, supporting rehabilitation and facilitating recovery over time.

## 1 Introduction

Mental illness and early cognitive decline have emerged as major global public health concerns in the 21st century (Santomauro et al. 2021; Rehm and Shield 2019), and the COVID‐19 pandemic further intensified the risks of mental and cognitive illness (Daly and Robinson 2022; Lakhan et al. 2024; Sousa et al. 2023; Percze et al. 2023). Beyond massive deaths and confirmed cases (World Health Organization 2026, 2024; Xiong et al. 2021; Alimohamadi et al. 2020; Chen et al. 2021; Zhou et al. 2020) COVID‐19 has also led to long‐term impairments, characterized by persistent symptoms and sequelae. This condition has come to be known as long COVID (Crook et al. 2021). According to the National Institute for Health and Care Excellence (NICE) (National Institute for Health and Care Excellence 2024) and the World Health Organization (WHO) (World Health Organization 2022), the syndrome is defined by symptoms lasting more than 3 months (12 weeks) after acute infection and that cannot be explained by another diagnosis.

Among the persistent symptoms, cognitive impairments, such as difficulty with memory and concentration, stand out as frequent complaints. Within this group, the phenomenon known as “brain fog” has gained attention. It refers to a set of neurological alterations resulting in mental confusion (Peter et al. 2022; Blomberg et al. 2021; Nalbandian et al. 2021; Aghajani 2024). Psychological disorders such as anxiety and depression, as well as sleep disturbances, also appear to be potentially long‐lasting and linked to COVID‐19, significantly affecting individuals' quality of life (Sousa et al. 2023; Percze et al. 2023).

While several reviews have examined long COVID, few have quantified the prevalence of psychological and cognitive sequelae using data from cohort studies, which allow stronger temporal inference. Understanding the frequency, duration and heterogeneity of these symptoms is essential for planning follow‐up and care strategies, as these conditions affect not only individual health but also social and economic domains—particularly through limitations in daily functioning and work capacity (Lakhan et al. 2024; Perez Giraldo et al. 2024; Azevedo et al. 2024; Shanbehzadeh et al. 2024).

Therefore, this systematic review and meta‐analysis was conducted to address this gap in the literature and to provide evidence on the long‐term mental health consequences of COVID‐19. Knowledge about these late‐onset impacts is crucial for nurses and other mental health professionals to design and implement care strategies aimed at prevention and rehabilitation across all levels of healthcare. At the managerial level, nursing plays a strategic role in planning, implementing and monitoring protocols and public policies to mitigate the psychosocial and cognitive effects of the disease.

## 2 Aim/Question

The aim of this review was to conduct a prevalence meta‐analysis of persistent psychological and cognitive impairments related to COVID‐19 based on cohort studies. The overarching research question was “What are the most frequent persistent psychological and cognitive sequelae resulting from SARS‐CoV‐2 infection as reported in cohort studies?”

## 3 Method

### 3.1 Study Design

This study is a systematic review with meta‐analysis, conducted in accordance with the PRISMA (Preferred Reporting Items for Systematic Reviews and Meta‐Analyses) guidelines (Page et al. 2021). The protocol was registered in the International Prospective Register of Ongoing Systematic Reviews (PROSPERO) under registration number CRD42023460632 on September 5, 2023.

### 3.2 Search Strategy

The search was conducted in the PubMed, Scopus, Embase, SciELO and Lilacs/BVS databases. A manual search of reference lists from the included articles and grey literature was also performed. Given the recent emergence of the topic and the aim of capturing the widest range of publications, no language restrictions were applied. The search timeframe was from January 2020 to November 2023, aligned with the onset of the COVID‐19 pandemic and the data collection period. Descriptors from MeSH and DeCS were used in combination with Boolean operators (AND, OR) to develop search strategies specific to each database (see Data S1).

The PECOs framework (Morgan et al. 2018) guided the search strategy: the Population (P) included adults; the Exposure (E) referred to infection by SARS‐CoV‐2; the Comparison (C) was not applicable; the Outcomes (O) included persistent psychological and cognitive impairments related to COVID‐19; and the Study design (s) focused on observational cohort studies.

### 3.3 Inclusion and Exclusion Criteria

Only cohort studies were included, as this was a prevalence meta‐analysis. Eligible studies had to include individuals with laboratory‐confirmed SARS‐CoV‐2 infection (via PCR or antigen testing) and assess persistent symptoms lasting at least 12 weeks after infection, in line with WHO and NICE definitions (National Institute for Health and Care Excellence 2024; World Health Organization 2022).

Studies based solely on clinical diagnosis or suspected cases without laboratory confirmation were excluded. Articles were also excluded if the follow‐up period was not clearly stated or did not meet the minimum 12‐week threshold. Studies involving exclusively paediatric populations were excluded. When data necessary for the meta‐analysis were not reported, authors were contacted via email; articles were excluded if no response was received within 1 month.

After full‐text assessment, studies that initially appeared to meet the inclusion criteria were excluded, for reasons such as: methodological limitations, same cohort for different studies and studies that showed symptoms unrelated to the psychological or cognitive domain.

### 3.4 Data Management

Search results were initially imported into EndNote to remove duplicates and then uploaded to Rayyan for screening. Two independent reviewers conducted the selection process. Discrepancies were resolved through consensus or consultation with a third reviewer. Inter‐rater agreement was assessed using the kappa statistic (McHugh 2012) during the abstract screening phase.

### 3.5 Data Analysis

Quantitative analyses were performed using the “meta” package (version 7.0–0) in R software (version 4.4.1). A pooled prevalence meta‐analysis with 95% confidence intervals (CI) was conducted for each outcome, using random‐effects models due to expected heterogeneity. For each symptom analysed, prevalence was calculated considering only the studies that included the outcome of interest. Thus, the meta‐analysis did not contain missing data, although losses were reported within each included article.

Heterogeneity was assessed using Cochran's Q test and the *I* ^2^ statistic, with significance set at *p* ≤ 0.05. Studies were categorized as homogeneous (*I* ^2^ ≤ 25%), moderately heterogeneous (*I* ^2^ > 25% and < 75%), or highly heterogeneous (*I* ^2^ ≥ 75%) (Higgins et al. 2003).

Subgroup analyses were performed based on follow‐up duration, cohort type, study continent, hospitalization status and methodological quality to explore sources of heterogeneity. Differences in prevalence between subgroups were considered significant at *p* < 0.05. The decision not to conduct a sensitivity analysis (i.e., excluding individual studies) was supported by the clinical and methodological variability observed across the included studies. Excluding individual studies was considered nonessential, given the level of heterogeneity already present in the populations analysed within each study.

The methodological quality of included studies was assessed using the adapted Newcastle–Ottawa Scale (NOS) for cohort studies (Wells et al. 2021), which evaluates sample selection, comparability, outcomes and follow‐up. Scores range from 0 to 9, classified as poor quality (0–3), fair quality (4–6), or good quality (7–9).

To assess publication bias, Egger's test and funnel plot were performed for each outcome. Results indicated moderate evidence of publication bias for anxiety (see Data S2), which was expected given the methodological heterogeneity of the included studies. The certainty of the evidence was not performed, since included studies were exclusively cohorts conducted in the pandemic context, with assumed high risk of bias.

## 4 Results

### 4.1 Study Selection

**FIGURE 1:**
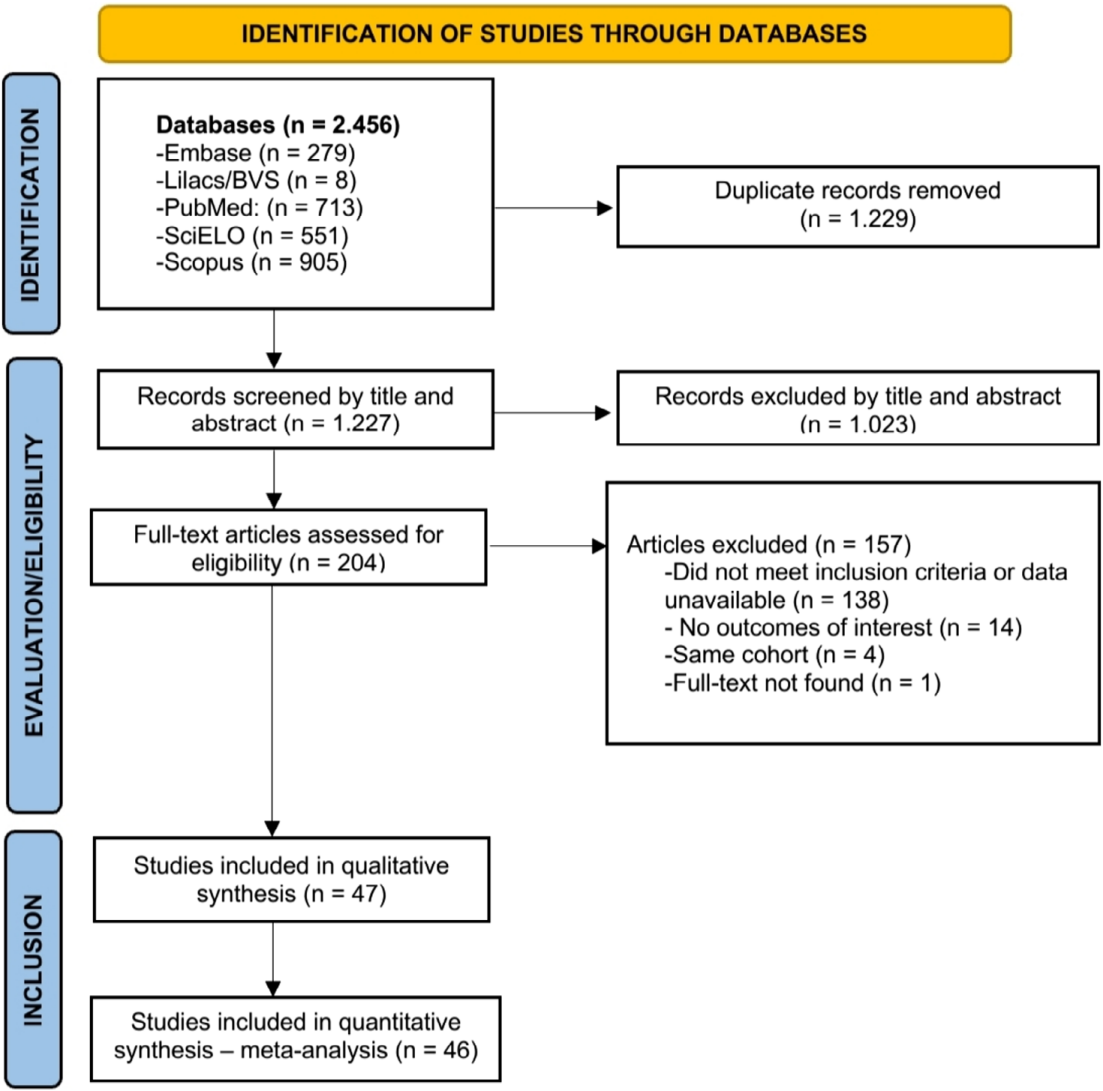
PRISMA framework.

This systematic review and meta‐analysis synthesized evidence from cohort studies evaluating the prevalence of persistent psychological and cognitive sequelae following COVID‐19 infection. A total of 47 studies were included in the systematic review, and 46 were retained in the meta‐analysis, as shown in the PRISMA framework below (Figure 1). No eligible studies were retrieved from grey literature. One article was excluded from the quantitative synthesis because it reported only one symptom not included in the defined outcomes (Strahm et al. 2022). The total sample size across the included studies was 192,158 participants. The inter‐rater agreement for abstract screening was substantial (Kappa = 0.792; *p* < 0.001) (McHugh 2012).

### 4.2 Characteristics of the Included Studies

The general characteristics of the included studies are presented in Table 1, including study cohort type, percentage of female participants, mean age, follow‐up duration after infection, sample size and methodological quality according to the Newcastle–Ottawa Scale (NOS).

**TABLE 1:** Studies' characteristics.

Identification	Cohort	Country	Sex (female)	Mean age	Follow‐up (months)	Sample (n)	NOS
González‐Ermosillo et al. (2021)	Prospective^†,‡,§,¶^	Mexico	34.6%	51.0	6	130	Fair
Malinowska et al. (2021)	Prospective^†,¶^	Poland	22.4%	53.0	6	67	Fair
Martinez et al. (2021)	Retrospective^†,¶^	Switzerland	75.4%	37.0	5,5	260	Fair
Och et al. (2021)	Prospective^†,‡^	Poland	50.6%	70.0	6	73	Fair
Ali et al. (2022)	Prospective^†,¶^	USA	73.1%	42.8	14,5	27	Fair
Asadi‐Pooja et al. (2022)	Prospective^†,¶^	Iran	45.3%	41.0	3	2.696	Fair
Buonsenso et al. (2022)	Prospective^†^	Italy	45.7%	44.6	7,5	107	Fair
Carazo et al. (2022)	Retrospective^¶^	Canada	77.2%	40.0	3	1.783	Fair
Desgranges et al. (2022)	Prospective^†,¶^	Switzerland	62.4%	41.0	5	418	Fair
Dryden et al. (2022)	Prospective^¶^	South Africa	51.3%	52.0	3	1.873	Fair
Estrada‐Codecido et al. (2022)	Retrospective^†,§,¶^	Canada	55.4%	38.0	3	466	Fair
Fang et al. (2022)	Prospective^‡^	China	52.1%	68.0	12	1.233	Fair
Fernández‐de‐las‐Peñas et al. (2022)	Prospective^¶^	Spain	46.5%	61.0	8,4	1.969	Fair
Förster et al. (2022)	Retrospective^§,¶^	Germany	56.5%	53.0	7,8	1.459	Fair
González et al. (2022)	Prospective^†,¶^	Spain	35.1%	61.0	12	97	Fair
Hastie et al. (2022)	Prospective^†,¶^	Scotland	63.6%	44.0	12	33.281	Fair
Kalak et al. (2022)	Prospective^¶^	Israel	50.0%	52.1	18	166	Fair
Marasco et al. (2022)	Prospective^‡,§^	Italy	40.7%	49.9	12	435	Fair
Pérez‐González et al. (2022)	Prospective^†,¶^	Spain	40.3%	57.0	6	248	Poor
Rigoni et al. (2022)	Prospective^†,¶^	Italy	36.1%	71.0	12	344	Fair
Rivera‐Izquierdo et al. (2022)	Prospective^†,‡,§,¶^	Spain	42.6%	61.2	12	453	Fair
Seeßle et al. (2022)	Prospective^†,‡,¶^	Germany	55.2%	57.0	12	96	Fair
Strahm et al. (2022)^a^	Prospective	Switzerland	82.2%	38.9	4	556	Good
Sun et al. (2022)	Retrospective^†,‡,§,¶^	China	55.2%	62.0	15	534	Fair
Takakura et al. (2022)	Retrospective^†,¶^	Japan	52.1%	35.8	3	257	Fair
Tartof et al. (2022)	Retrospective^†,‡^	USA	53.7%	41.2	5	127.859	Good
Titze‐de‐Almeida et al. (2022)	Prospective^†,‡,§,¶^	Brazil	61.0%	41.2	6,5	236	Fair
Visconti et al. (2022)	Prospective^‡^	Brazil	52.3%	60.5	12	88	Fair
Wisnivesky et al. (2022)	Prospective^†,‡,§^	USA	64.9%	50.1	6	453	Fair
Zuschlag et al. (2022)	Retrospective^†,‡,§,¶^	Germany	45.7%	62.0	12	162	Fair
Ballouz et al. (2023)	Prospective^†,¶^	Switzerland	49.6%	50.0	6	504	Good
Brunvoll et al. (2023)	Prospective^¶^	Norway	71.3%	48.0	9	1.420	Good
D'Ávila et al. (2023)	Prospective^†^	Brazil	80.6%	42.2	6	174	Fair
Fumis et al. (2023)	Retrospective^‡,§^	Brazil	33.3%	59.9	3	1.122	Fair
Gentilotti et al. (2023)	Prospective^¶^	France, Italy, Argentina, Spain and Netherlands	43.4%	57.2	12	1.796	Fair
Guillen‐Burgos et al. (2024)	Prospective^†,‡,§^	Colombia	49.7%	51.5	24	1.565	Fair
Hyassat et al. (2023)	Prospective^†,¶^	Jordan	70.7%	34.4	12	140	Fair
Klinkhammer et al. (2023)	Prospective^†,‡,§,¶^	Netherlands	30.2%	64.0	9	205	Fair
Krishnadath et al. (2023)	Prospective^†,§,¶^	Suriname	62.3%	49.0	3,5	106	Fair
Lemhöfer et al. (2023).	Prospective^†^	Germany	66.4%	49.5	9,6	952	Fair
Monteiro et al. (2023)	Prospective^†,¶^	Brazil	69.8%	41.4	9	48	Fair
Ranucci et al. (2023)	Prospective^†,‡,§,¶^	Italy	33.9%	63.1	17	121	Fair
Serrano et al. (2023)	Prospective^‡,§,¶^	Colombia	29.6%	62.0	12	135	Fair
Silva et al. (2023)	Prospective^¶^	Braziç	63.9%	38.4	12	69	Fair
Smith et al. (2023)	Prospective^†,¶^	Belgium	64.6%	43.1	3	5.727	Fair
Steinmetz et al. (2023)	Prospective^†,‡,§,¶^	Germany	78.5%	48.2	6	104	Fair
Sykes et al. (2023)	Retrospective^†,‡,¶^	England	37.5%	62.0	3	144	Fair
Total						192.158	

*Note:* Sequelae: †, anxiety; ‡, cognitive impairments; §, sleep disturbances; ¶, depression.

^a^ Study/outcome not included in the meta‐analysis.

### 4.3 Persistent Psychological and Cognitive Sequelae

**FIGURE 2:**
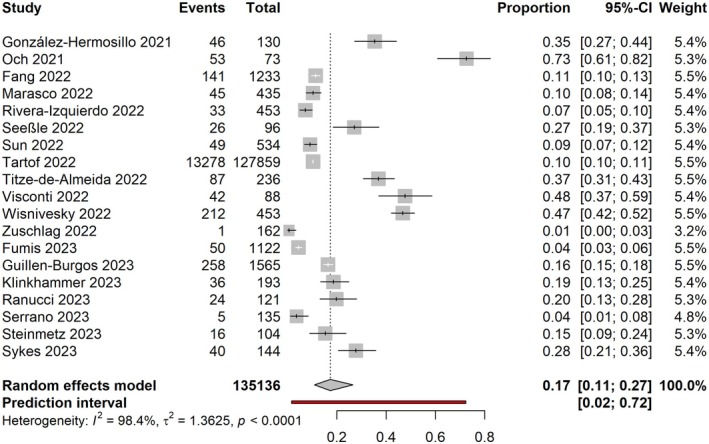
Forest plot anxiety.

Anxiety was the most prevalent symptom (0.1740; 95% CI 0.1095–0.2652, Figure 2), followed by cognitive impairments (0.1520; 95% CI 0.1110–0.2047, Figure 3), sleep disturbances (0.1353; 95% CI 0.0925–0.1937, Figure 4) and depression (0.1094; 95% CI 0.0619–0.1861, Figure 5; see Data S3).

**FIGURE 3:**
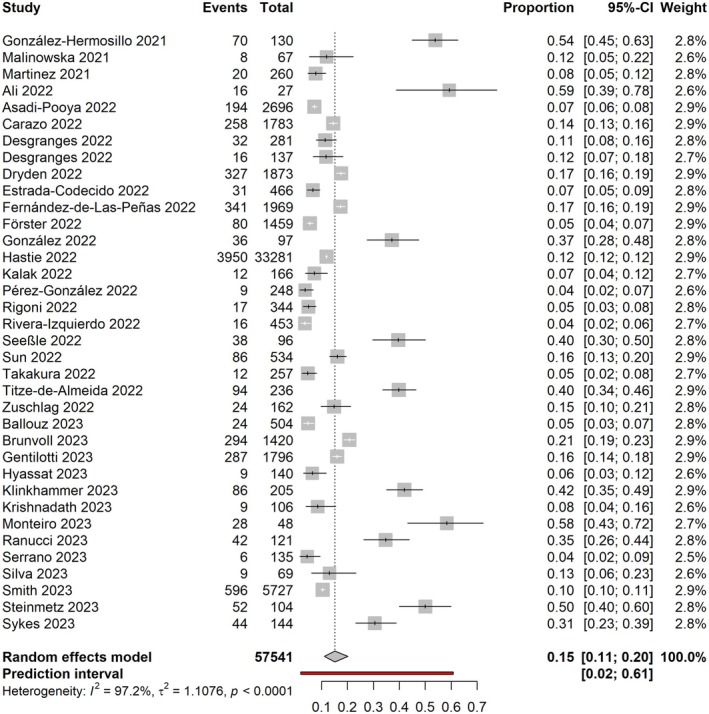
Forest plot of cognitive impairments.

**FIGURE 4:**
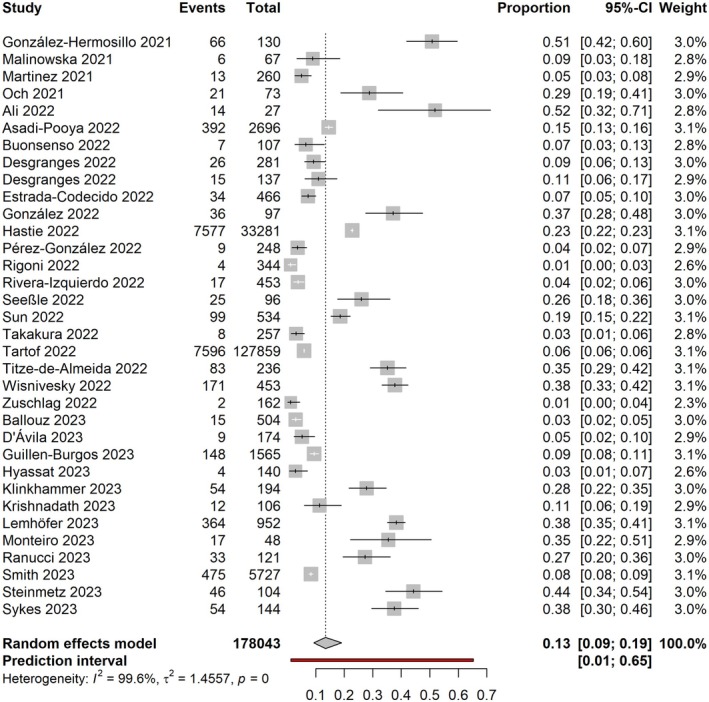
Forest plot sleep disturbances.

**FIGURE 5:**
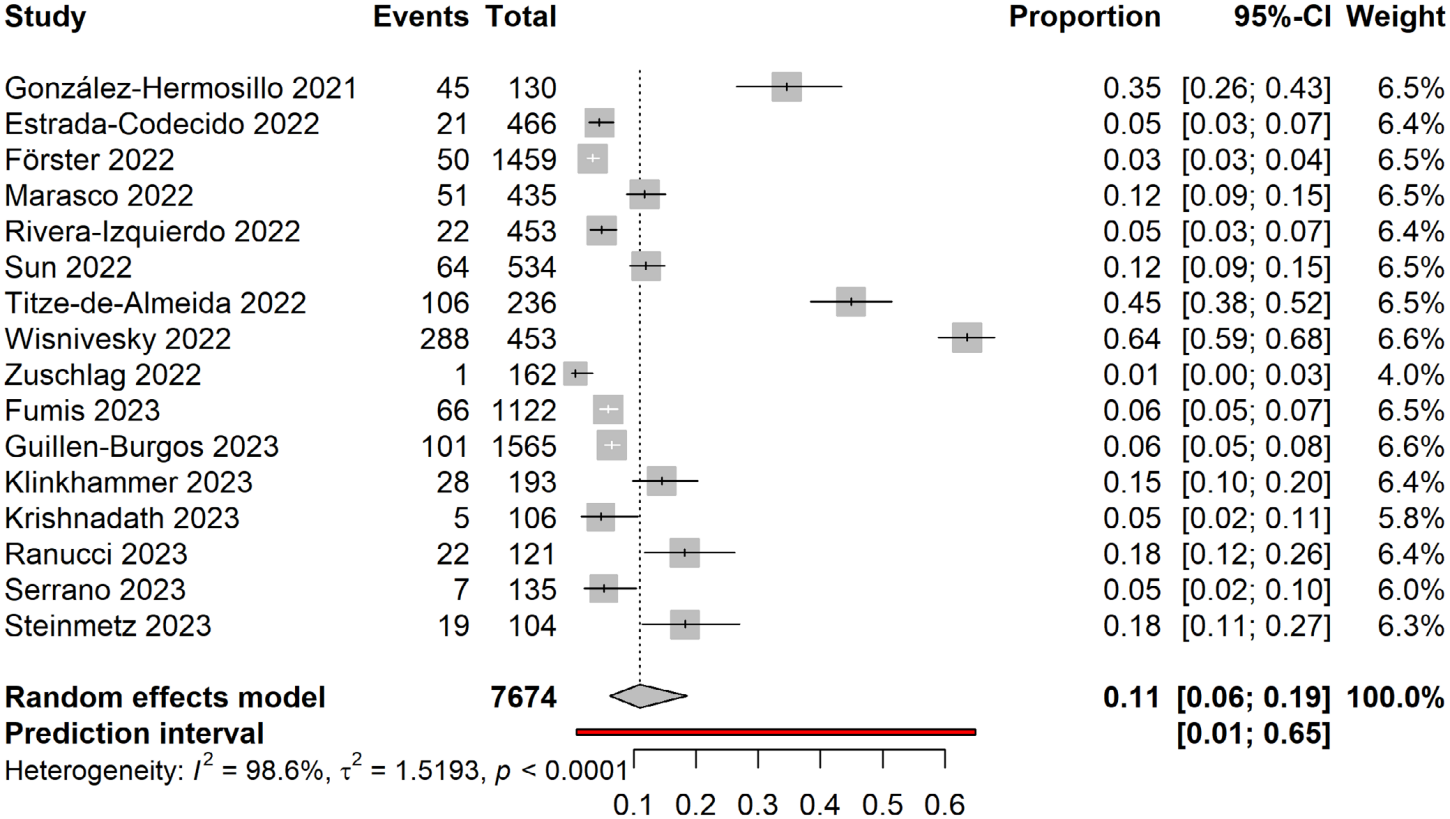
Forest plot depression.

Although this meta‐analysis focused on the most frequent outcomes, other relevant conditions were noted in the full‐text articles, including burnout (Strahm et al. 2022), post‐traumatic stress disorder (PTSD) (Wisnivesky et al. 2022; Zuschlag et al. 2022; Fumis et al. 2023; Klinkhammer et al. 2023), irritability (Monteiro et al. 2023; Steinmetz et al. 2023), apathy (Steinmetz et al. 2023) and anomia/aphasia (Seeßle et al. 2022; Gentilotti et al. 2023).

### 4.4 Subgroup Analyses

Subgroup analyses (see Data S4) were conducted to explore sources of heterogeneity. For depression, follow‐up durations of less than 6 months showed the lowest heterogeneity; for anxiety, moderate heterogeneity was observed in studies from Asia. However, due to the small number of studies in these groups, these findings should be interpreted cautiously.

A statistically significant difference in depression prevalence was observed by follow‐up duration, with higher rates reported between 6 and 12 months (0.13; 95% CI 0.06–0.28). Hospitalization status showed a significant effect only for anxiety. However, most studies included hospitalized participants and none focused solely on non‐hospitalized individuals.

Significant differences in prevalence were observed by cohort type, with higher anxiety and depression rates reported in prospective cohorts. While subgroup differences in cognitive and sleep outcomes were associated with study quality, most included studies were rated as fair quality, limiting interpretation.

Regarding geographic location, cognitive impairments were significantly more prevalent in studies conducted in North America (0.27; 95% CI 0.08–0.61) and South America (0.19; 95% CI 0.06–0.44).

## 5 Discussion

The results of this study point to anxiety, cognitive impairments, sleep disturbances and depression as the main persistent psychological and cognitive sequelae of COVID‐19.

The findings of this systematic review demonstrate that psychological and cognitive impairments persisted beyond the acute phase of COVID‐19 infection. These conditions represent a public health concern, as they not only affect individual health but also have broader social and economic consequences, considering their impact on the ability to perform basic activities of daily living (Perez Giraldo et al. 2024; Azevedo et al. 2024). Additionally, the pandemic caused economic disruptions due to social distancing measures, which altered work patterns—especially for workers with precarious employment—potentially exacerbating mental health problems among economically vulnerable populations (Matsubayashi et al. 2022).

Psychological symptoms such as anxiety and depression were frequently reported both during and after the COVID‐19 infection (Sousa et al. 2023; Marchi et al. 2023; Seighali et al. 2024; Tang et al. 2022). The widespread fear related to the pandemic, uncertainty about its course and the need for social restrictions contributed significantly to the development of these symptoms (Bäuerle et al. 2020; Jung et al. 2020). Additionally, sleep disturbances—such as insomnia or excessive daytime sleepiness, which affect both physical and mental well‐being—were also commonly reported (Percze et al. 2023).

Another systematic review reported even higher pooled prevalence estimates for psychological impairments (Seighali et al. 2024). However, it is important to note that prevalence estimates for anxiety and depression may be overestimated due to the short‐term nature of mental health changes during the pandemic, which were not always accounted for in the studies and the likely decline in these symptoms over time (Daly and Robinson 2022). Differences in prevalence rates across reviews may be attributed to varying inclusion criteria, such as study type and data collection methods.

Prediction intervals were wide for all outcomes (e.g., 2%–73% for anxiety). This substantial heterogeneity indicates that prevalence in individual populations may differ significantly from the pooled estimates. Mental health nurses should screen for the presence of these symptoms in the pre‐COVID clinical history in order to determine whether they may be related to long COVID, since wide intervals may reflect a lack of association with prior psychological and cognitive symptoms.

The asymmetry observed in the funnel plot for anxiety suggests a possible underrepresentation of studies with smaller samples and lower prevalence estimates, indicating that the pooled estimate of 17% may be overestimated and should be interpreted with caution. While this aspect should be taken into account, it is also important to consider the rising levels of anxiety in the general population as an ongoing public health issue, which has been widely reported in the literature.

The peak in depression observed between 6 and 12 months after infection revealed a pattern of worsening prior to improvement, a finding that may be related not only to depression itself but also to other long COVID sequelae, which often overlap and hinder the return to usual daily and work routines. This condition may help explain the increase in depressive symptoms and the delay in recovery, as the chronic nature of persistent symptoms contributes to psychological distress.

Mental health nurses should take into account the continued presence of long COVID sequelae when screening for depression, as these symptoms influence the patient's socioemotional coping. This suggests that the recovery trajectory is not linear and indicates that mental health screening should continue beyond the initial phase, particularly during medium‐term follow‐up. Therefore, reassessment should occur at a minimum at 6 and 12 months after the acute illness.

Regarding cognitive impairments, memory disturbances have been widely recognized as a common long COVID symptom (Shanbehzadeh et al. 2024). Furthermore, difficulties with concentration, attention and anomia have also been observed during the post‐acute phase (Perez Giraldo et al. 2024; Herrera et al. 2023). One study suggests that “brain fog” may be linked to neural network damage in patients with long COVID sequelae (Nouraeinejad 2023); however, this is challenged by a 2024 study showing that neuropsychiatric symptoms and cognitive deficits are not the result of ongoing neural injury (Taquet et al. 2024) and by a systematic review concluding that although the condition may be associated with brain abnormalities, a causal relationship cannot yet be established. These discrepancies across the evidence suggest that the symptoms should not be interpreted as permanent neurological damage. This highlights the importance of nurses promoting rehabilitation strategies and activities that support patients in their neurological recovery.

A meaningful interpretation of the regional differences requires considering that the United States has the highest absolute number of COVID‐19 infections (World Health Organization 2026), which may help explain the prominence of persistent cognitive sequelae in that population. In addition, the higher prevalence of cognitive sequelae reported in studies from the United States may reflect differences in assessment methods, access to post‐COVID follow‐up and sociocultural factors that shape how these sequelae are recognized and reported. For mental health nursing practice, the assessment of cognitive symptoms should take into account the context and specific characteristics of the care setting, avoiding the direct application of global estimates.

Several studies (González‐Ermosillo et al. 2021; Martinez et al. 2021; Dryden et al. 2022; Förster et al. 2022; Hastie et al. 2022; Marasco et al. 2022; Seeßle et al. 2022; Lemhöfer et al. 2023; Ranucci et al. 2023; Steinmetz et al. 2023) reported the presence of pre‐existing mental disorders among participants, with prevalence rates ranging from 1.0% to 27.3%. Evidence suggests that pre‐existing psychiatric conditions may contribute to the onset or worsening of symptoms associated with long COVID (Xiong et al. 2021; Miguel‐Puga et al. 2021; Milde et al. 2023). The wide variation in pre‐existing mental health conditions across studies raises the question of whether these are new symptoms or an exacerbation of previous problems. The implications of these findings for assessment and treatment require screening the baseline mental health status of patients who experienced COVID‐19 infection. Measuring persistent sequelae requires examining patients' pre‐pandemic mental health history, as well as considering their clinical course related to COVID‐19.

Individuals with persistent psychological impairments in the post‐COVID period often face social stigma, which can hinder their recovery by discouraging them from seeking treatment. This can lead to increased suffering, higher levels of anxiety and depression and a reduced quality of life (Scholz et al. 2023; Yuan et al. 2021). It is therefore essential to develop and implement public health education strategies aimed at reducing the stigma surrounding long COVID and encouraging those affected to seek the care necessary to improve their mental health and overall well‐being (Kang et al. 2021).

Given the complexity of persistent psychological and cognitive impairments following COVID‐19 infection, and the current lack of concrete evidence on their nature and causes, it is essential to expand research efforts for long‐term monitoring of affected individuals. This will help clarify the long‐term consequences, causal relationships and broader impacts of these conditions (Nouraeinejad 2023).

Despite differences in prevalence estimates across some studies, no sensitivity analyses were conducted. *Outliers* included results such as Och (Page et al. 2021) reporting 73% depression and Gonzalez‐Hermosillo (González‐Ermosillo et al. 2021) reporting 51% cognitive impairment, values higher than the pooled meta‐analytic estimates. These differences may reflect disparities in study population size, regional variations related to culture, surveillance strategies and access to health services, as well as differences in analytical methods. Moreover, the variation in sample size between studies (e.g., 130 participants in González‐Ermosillo et al. (2021) and 127,859 in Tartof et al. (2022)) does not directly determine each study's weight in the meta‐analysis, as shown in the forest plots (Figures 2, 3, 4, 5). Thus, although mega‐studies do not dominate the pooled estimates, their influence should still be taken into account, particularly given the high heterogeneity across studies.

Although the heterogeneity observed is a limitation of this study, it should not be viewed solely as such. On the contrary, it highlights that the prevalence of persistent symptoms varies considerably across contexts, populations and measurement methods, indicating that there is no single prevalence applicable to all settings. Even after subgroup analyses, this heterogeneity remains; however, the findings continue to be clinically meaningful when interpreted as population‐level risk indicators rather than precise individual outcomes. Therefore, the results are important for clinical practice, as they suggest that persistent symptoms may be commonly encountered during follow‐up.

In this study, using cohort data collected during the pandemic period, heterogeneity is an important finding, as it reflects the real diversity of the mental health burden following COVID‐19 infection. When evaluating patients in clinical practice, mental health nurses should remain attentive to complaints related to anxiety, cognitive impairments, sleep and depression, and to their possible connection with psychological and cognitive sequelae of COVID‐19. It is important to investigate how the pandemic period may have affected the patient's clinical history, as considering this broader context allows for a more comprehensive assessment that can better inform nursing interventions.

The persistence of symptoms raises significant concerns for clinical practice due to the chronic nature of the condition, which requires nurses to have the skills to assess and detect anxiety, cognitive and sleep disturbances and depression. This study suggests that mental health nurses need enhanced knowledge regarding treatment and mitigation of risks associated with health complications and reduced quality of life. The continued presence of these symptoms increases the burden on public health. Another point of concern arising from these findings is that most studies include individuals who are economically active, underscoring the need for the implementation of return‐to‐work programs capable of supporting people with post‐COVID health conditions.

## 6 Strengths and Limitations

The study is relevant due to its simultaneous analysis of multiple outcomes, which enables the identification of those with the highest prevalence, as well as the breadth and robustness of its sample.

Its main limitation is the high heterogeneity observed in the studies, even after subgroup analyses, reflecting the wide variation in the prediction intervals of the analysed outcomes, which may be related to the diversity of data collection methods—although this reflects a common feature of pandemic‐related studies. The decision to perform the meta‐analysis despite the high heterogeneity (*I* ^2^ ranging from 97% to 100%) was based on the fact that anxiety, cognitive impairments, sleep disturbances and depression are common public health problems in the general population, and the substantial variability identified in this study uniquely reflects the effects of the pandemic. The initiative to provide central prevalence estimates is supported by the high occurrence of these symptoms in contexts prior to the COVID‐19 pandemic. In this sense, the findings highlight the distinct reality of studies that sought to investigate long COVID sequelae. However, we acknowledge that the pooled results should be interpreted with caution, as individual values may vary across studies. The meta‐analysis was complemented by prediction interval analyses and a discussion of potential sources of heterogeneity, ensuring that the synthesis does not rely on a single number but instead reflects the diversity of contexts represented.

In addition, the lack of a control group of non‐infected individuals in most studies limits the ability to draw definitive conclusions about the association between these persistent symptoms and SARS‐CoV‐2 infection, given that all people experienced social and economic impacts during the pandemic. Therefore, in clinical practice, mental health complaints should be analysed in relation to the patient's account of their previous experiences, using a clinical interview that explores the different biopsychosocial dimensions affected both by the pandemic period and by the illness resulting from SARS‐CoV‐2 infection.

## 7 Implications and Recomemmendations

This study reinforces the importance of longitudinal monitoring of individuals with persistent COVID‐19 sequelae. In clinical practice, it is recommended that mental health nurses perform systematic screening of psychological and cognitive sequelae using validated instruments, with periodic reassessments throughout follow‐up.

Outpatient rehabilitation programs with a cognitive‐behavioural approach may be effective interdisciplinary strategies for enabling mental health nurses to provide safe and high‐quality care to patients with long COVID sequelae. Interventions such as psychoeducation, empathic listening and guidance on self‐care may be sufficient for some individuals, while moderate and severe cases may require psychotherapeutic follow‐up and pharmacological treatment. In light of limited funding for specialized services, primary care plays a key role in the continuous follow‐up of these patients, with interdisciplinary collaboration according to identified needs. Return to work protocols for individuals experiencing long COVID sequelae can support nurses working in different care settings in monitoring and preventing complications. Additionally, the development of quick‐reference materials for healthcare professionals may assist in clinical decision‐making when dealing with the main conditions associated with long COVID. Nurse managers and leaders also play a central role in organizing care pathways, implementing monitoring protocols and advocating for policies that support long‐term care.

## 8 Relevance to Mental Health Nursing

The long COVID condition has implications for mental health nursing practices due to the high prevalence of psychological and cognitive sequelae resulting from COVID‐19 infection in the population, making it a public health issue with economic and social impacts. This systematic review with meta‐analysis examined cohort studies, providing evidence based on 192,158 participants and identifying anxiety as the most prevalent symptom, followed by cognitive impairments, sleep disturbances and depression. Mental health nurses could incorporate these aspects into their practice when working with patients experiencing persistent health deterioration following COVID‐19 infection.

## 9 Conclusion

This systematic review investigated the prevalence of psychological and cognitive impairments in the post‐COVID‐19 period, offering a comprehensive overview of the diverse ways in which these conditions manifest. Although the findings indicate high prevalence rates of these impairments in cohort studies, considerable variation was observed across the studies. The high heterogeneity, even after subgroup analyses, reflects a frequent scenario in pandemic‐related research, likely related to variations in data collection methods.

Considering that these impairments represent a public health issue with broad implications, there is a clear need for the development of public policies and support strategies to mitigate these consequences.

## Funding

This study is linked to a research project funded by a productivity grant under process number 312850/2025‐5. Modality/Level: PQ‐C. CNPq Call No. 18/2024.

## Ethics Statement

As this study is a systematic review and meta‐analysis of previously published data, ethical approval was not required in accordance with the guidelines of the Journal of Psychiatric and Mental Health Nursing.

## Consent

The authors have nothing to report.

## Conflicts of Interest

The authors declare no conflicts of interest.

## Supporting information


**Data S1:** Search strategies according to database.

**Data S2:** Egger's test and funnel plot.

**Data S3:** Pooled prevalence of psychological and cognitive sequelae, prediction interval and heterogeneity.

**Data S4:** Subgroup analysis for psychological and cognitive sequelae.

## Data Availability

The datasets generated and/or analysed during the current study are available from the corresponding author upon reasonable request.
