# Supplementary material for: Psychological and Cognitive Sequelae of COVID‐19: Systematic Review and Meta‐Analysis
Source: J Psychiatr Ment Health Nurs. 2026 May 8;33(4):653–66. doi: 10.1111/jpm.70139 (PMC13341033; doi:10.1111/jpm.70139)
Supplement: Supplementary file 1 — Data S1: Search strategies according to database. [file JPM-33-653-s004.docx]

**Supplementary 1 - Search strategies according to database.**

| **Database** | **Search strategy** |
| --- | --- |
| **PubMed** | (("Post-Acute COVID-19 Syndrome"[Mesh]) OR (COVID-19 Syndrome, Post-Acute[Text Word]) OR (Post-Acute COVID-19 Syndromes[Text Word]) OR (Long Haul COVID-19[Text Word]) OR (COVID-19, Long Haul[Text Word]) OR (Long Haul COVID 19[Text Word]) OR (Long Haul COVID-19s[Text Word]) OR (Post Acute COVID-19 Syndrome[Text Word]) OR (Post Acute COVID 19 Syndrome[Text Word]) OR (Long COVID[Text Word]) OR (Post- Acute Sequelae of SARS-CoV-2 Infection[Text Word]) OR (Post Acute Sequelae of SARS CoV 2 Infection[Text Word]) OR (Post-COVID Conditions[Text Word]) OR (Post COVID Conditions[Text Word]) OR (Post-COVID Condition[Text Word]) OR (Long-Haul COVID[Text Word]) OR (Long Haul COVID[Text Word]) OR (COVID, Long-Haul[Text Word]) OR (Long-Haul COVIDs[Text Word])) AND (("Cohort Studies"[Mesh]) OR (Cohort Study[Text Word]) OR (Studies, Cohort[Text Word]) OR (Study, Cohort[Text Word]) OR (Concurrent Studies[Text Word]) OR (Studies, Concurrent[Text Word]) OR (Concurrent Study[Text Word]) OR (Study, Concurrent[Text Word]) OR (Closed Cohort Studies[Text Word]) OR (Cohort Studies, Closed[Text Word]) OR (Closed Cohort Study[Text Word]) OR (Cohort Study, Closed[Text Word]) OR (Study, Closed Cohort[Text Word]) OR (Studies, Closed Cohort[Text Word]) OR (Birth Cohort Studies[Text Word]) OR (Birth Cohort Study[Text Word]) OR (Cohort Studies, Birth[Text Word]) OR (Cohort Study, Birth[Text Word]) OR (Studies, Birth Cohort[Text Word]) OR (Study, Birth Cohort[Text Word]) OR (Analysis, Cohort[Text Word]) OR (Analyses, Cohort[Text Word]) OR (Cohort Analyses[Text Word]) OR (Cohort Analysis[Text Word]) OR (Historical Cohort Studies[Text Word]) OR (Cohort Studies, Historical[Text Word]) OR (Cohort Study, Historical[Text Word]) OR (Historical Cohort Study[Text Word]) OR (Study, Historical Cohort[Text Word]) OR (Studies, Historical Cohort[Text Word]) OR (Incidence Studies[Text Word]) OR (Incidence Study[Text Word]) OR (Studies, Incidence[Text Word]) OR (Study, Incidence[Text Word])) |
| **Scopus** | ( TITLE-ABS-KEY ( "COVID-19 Syndrome, Post-Acute" OR "Post-Acute COVID-19 Syndromes" OR "Long Haul COVID-19" OR "COVID-19, Long Haul" OR " Long Haul COVID 19" OR "Long Haul COVID-19s" OR "Post Acute COVID-19 Syndrome" OR "Post Acute COVID 19 Syndrome" OR "Long COVID" OR "Post-Acute Sequelae of SARS- CoV-2 Infection" OR "Post Acute Sequelae of SARS CoV 2 Infection" OR "Post-COVID Conditions" OR "Post COVID Conditions" OR "Post-COVID Condition" OR "Long-Haul COVID" OR "COVID, Long-Haul" OR "Long Haul COVID" OR "Long-Haul COVIDs" OR "Post-Acute COVID-19 Syndrome" ) AND TITLE-ABS-KEY ( "Cohort Study" OR "Studies, Cohort" OR "Study, Cohort" OR "Concurrent Studies" OR "Studies, Concurrent" OR "Concurrent Study" OR "Study, Concurrent" OR "Closed Cohort Studies" OR "Cohort Studies, Closed" OR "Closed Cohort Study" OR "Cohort Study, Closed" OR "Study, Closed Cohort" OR "Studies, Closed Cohort" OR "Birth Cohort Studies" OR "Birth Cohort Study" OR "Cohort Studies, Birth" OR "Cohort Study, Birth" OR "Studies, Birth Cohort" OR "Study, Birth Cohort" OR "Analysis, Cohort" OR "Analyses, Cohort" OR "Cohort Analyses" OR "Cohort Analysis" OR "Historical Cohort Studies" OR "Cohort Studies, Historical" OR "Cohort Study, Historical" OR "Historical Cohort Study" OR "Study, Historical Cohort" OR "Studies, Historical Cohort" OR "Incidence Studies" OR "Incidence Study" OR "Studies, Incidence" OR "Study, Incidence" OR "Cohort Studies" ) ) |
| **Embase** | (('long covid'/exp OR 'chronic covid syndrome' OR 'covid long-hauler' OR 'covid-19 long-hauler' OR 'long haul covid' OR 'long haul covid-19' OR 'long hauler covid' OR 'post covid 19 fatigue' OR 'post covid 19 neurological syndrome' OR 'post covid 19 syndrome' OR 'post covid fatigue' OR 'post covid impairment' OR 'post covid syndrome' OR 'post-acute covid syndrome' OR 'post-acute covid-19') AND 'post-acute covid-19 fatigue' OR 'post-acute covid-19 neurological syndrome' OR 'post-acute covid-19 syndrome' OR 'post-acute sequelae of sars-cov-2 infection' OR 'post-covid condition' OR 'post-covid-19 condition') AND ('cohort analysis'/exp OR 'analysis, cohort' OR 'cohort fertility' OR 'cohort life cycle' OR 'cohort studies' OR 'cohort study' OR 'fertility, cohort' OR 'cohort analysis') |
| **SciELO** | ((mh:"Cohort studies" OR "Cohort studies" OR "Cohort Study" OR "Estudo de coorte" OR "Estudio de cohorte" OR mh:"Follow-up studies" OR "Follow-up studies" OR "Estudos de Follow-up" OR "Estudios de Seguimiento" OR "Estudios de Follow-up" OR mh:"Longitudinal studies" OR "Longitudinal studies" OR "Estudos Longitudinais" OR "Estudios Longitudinales" OR "Longitudinal Survey" OR "Longitudinal Study" OR "Longitudinal Surveys" OR mh:"Prospective studies" OR "Prospective studies" OR "Estudos Prospectivos" OR "Estudios Prospectivos" OR "Prospective Study" OR mh:"Retrospective studies" OR "Retrospective Studies" OR "Estudos Retrospectivos" OR "Estudios Retrospectivos" OR "Retrospective Study" OR ti:cohort* OR ti:coorte* OR ti:longitudina* OR ti:prospectiv* OR ti:retrospectiv* OR "Estudos de Incidência" OR "Estudios de Concurrencia" OR "Estudios de Incidencia" OR "Incidence Studies" OR "Incidence Study" OR "Concurrent Studies" OR "Concurrent Study")) AND (("Síndrome Pós- COVID-19 Aguda" OR "Post-Acute COVID-19 Syndrome" OR "Síndrome Post Agudo de COVID-19" OR "Afecções Pós-COVID" OR "COVID Longa" OR "COVID de Longo Curso" OR "Sequela Pós-Infecção por SARS-CoV-2 Aguda" OR "COVID-19" OR "COVID19" OR "Infecção pelo SARS-CoV-2")) |
| **Lilacs/ BVS** | (mh:(Síndrome Pós-COVID-19 Aguda)) OR (mh:(Post-Acute COVID-19 Syndrome)) OR (mh:(Síndrome Post Agudo de COVID-19)) OR (Afecções Pós-COVID) OR (COVID Longa) OR (COVID de Longo Curso) OR (Sequela Pós-Infecção por SARS-CoV-2 Aguda) AND (mh:(Estudos de Coortes)) OR (mh:(Cohort Studies)) OR (mh:(Estudios de Cohortes)) OR (Análise de Coortes) OR (Estudo de Coorte de Nascimento) OR (Estudos de Coortes de Nascimentos) OR (Estudos de Incidência) OR (Estudos Fechados de Coortes) OR (Estudos Históricos de Coortes) |

Source: Authors (2025)
