# Supplementary material for: Psychological and Cognitive Sequelae of COVID‐19: Systematic Review and Meta‐Analysis
Source: J Psychiatr Ment Health Nurs. 2026 May 8;33(4):653–66. doi: 10.1111/jpm.70139 (PMC13341033; doi:10.1111/jpm.70139)

**Supplementary 2 - Egger’s Test and Funnel plot**

The data below present the results of Egger’s test and the funnel plot for each outcome evaluated.

**Anxiety**

Test result: t = 2.52, df = 17, p-value = 0.0220

Bias estimate: 4.4346 (SE = 1.7593)


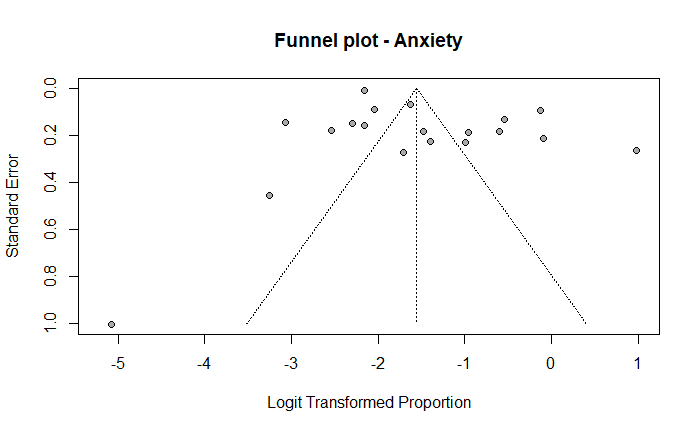


**Cognitive impairments**

Test result: t = 1.52, df = 34, p-value = 0.1371

Bias estimate: 1.9640 (SE = 1.2898)


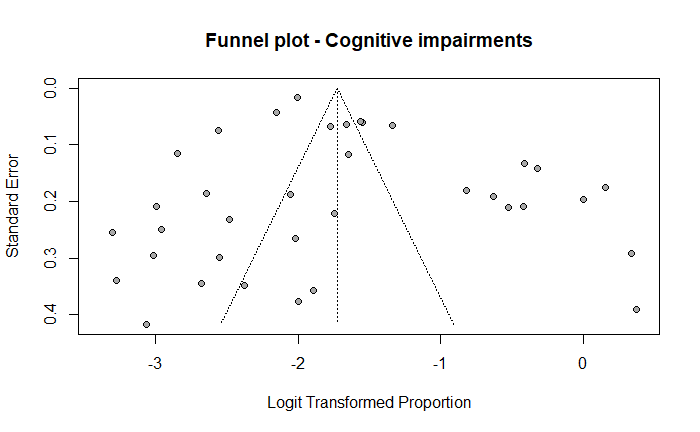


**Sleep disturbances**

Test result: t = 0.81, df = 32, p-value = 0.4265

Bias estimate: 2.6796 (SE = 3.3271)


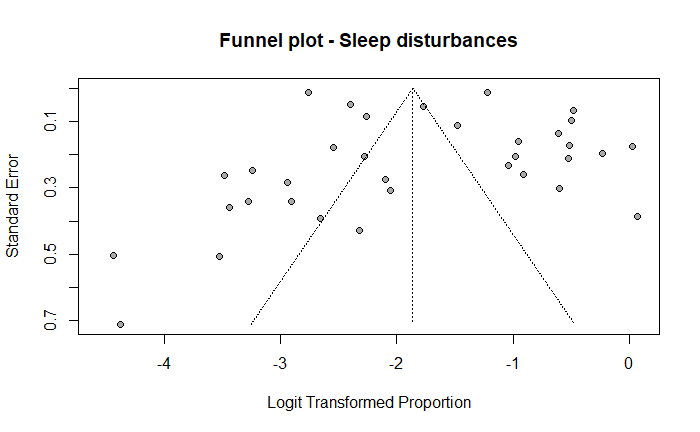


**Depression**

Test result: t = -1.16, df = 14, p-value = 0.2638

Bias estimate: -5.9626 (SE = 5.1214)


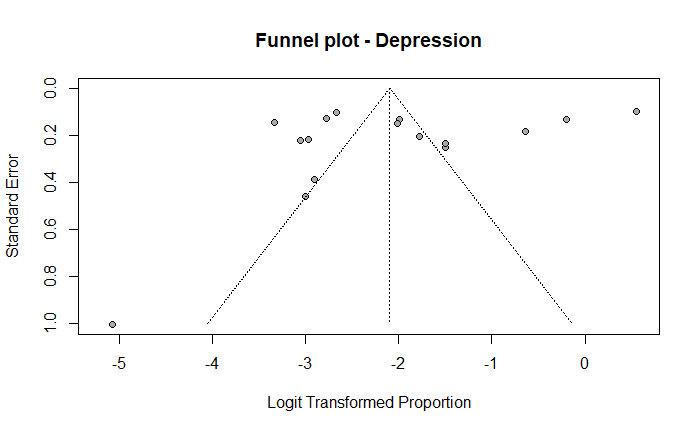

Supplement: Supplementary file 2 — Data S2: Egger's test and funnel plot. [file JPM-33-653-s001.docx]
