# Supplementary material for: Psychological and Cognitive Sequelae of COVID‐19: Systematic Review and Meta‐Analysis
Source: J Psychiatr Ment Health Nurs. 2026 May 8;33(4):653–66. doi: 10.1111/jpm.70139 (PMC13341033; doi:10.1111/jpm.70139)
Supplement: Supplementary file 3 — Data S3: Pooled prevalence of psychological and cognitive sequelae, prediction interval and heterogeneity. [file JPM-33-653-s002.docx]

**Supplementary 3 - Pooled Prevalence of Psychological and Cognitive Sequelae, Prediction Interval, and Heterogeneity.**

| Outcome | Studies | Events / Observations | Prevalence (95% CI) | Prediction Interval  (95% CI) | I² (%) | p-value |
| --- | --- | --- | --- | --- | --- | --- |
| Anxiety | 19 | 14,442 / 135,136 | 0.1740 (0.1095–0.2652) | (0.0165–0.7257) | 98.4 | <0.001 |
| Cognitiveᵃ | 35 | 7,173 / 57,541 | 0.1520 (0.1110–0.2047) | (0.0194–0.6188) | 97.3 | <0.001 |
| Sleep | 33 | 17,452 / 178,043 | 0.1353 (0.0925–0.1937) | (0.0122–0.6646) | 99.7 | <0.001 |
| Depression | 16 | 896 / 7,674 | 0.1094 (0.0619–0.1861) | (0.0080–0.6532) | 98.6 | <0.001 |

**CI:** Confidence Interval; **I²:** Higgins' inconsistency index (heterogeneity).
ᵃCognitive sequelae include memory, attention, confusion, concentration, brain fog, or studies reporting grouped cognitive symptoms.
