# Supplementary material for: Psychological and Cognitive Sequelae of COVID‐19: Systematic Review and Meta‐Analysis
Source: J Psychiatr Ment Health Nurs. 2026 May 8;33(4):653–66. doi: 10.1111/jpm.70139 (PMC13341033; doi:10.1111/jpm.70139)
Supplement: Supplementary file 4 — Data S4: Subgroup analysis for psychological and cognitive sequelae. [file JPM-33-653-s003.docx]

**Supplementary 4 - Subgroup Analysis for Psychological and Cognitive Sequelae.**

| **Outcomes/Subroups** | **Anxiety** | | | **Cognitive impairments** | | | | | | **Sllep disturbances** | | | | | | | **Depression** | | | | |
| --- | --- | --- | --- | --- | --- | --- | --- | --- | --- | --- | --- | --- | --- | --- | --- | --- | --- | --- | --- | --- | --- |
|  | **Studies/ Pop.^a^** | **Prevalence**  **(IC 95%)** | **I²** | **Studies/ Pop.^a^** | | | **Prevalence**  **(IC 95%)** | | **I²** | **Studies/ Pop.^a^** | **Prevalence**  **(IC 95%)** | **I²** | | | | | **Studies / Pop.^a^** | **Prevalence**  **(IC 95%)** | | | **I²** |
| **Follow-up period** |  |  |  |  | | |  | |  |  |  |  | | | | |  |  | | |  |
| < 6 months | 3 / 129.125 | 0,11 (0,04–0,29) | 97,5% | 10 / 13.730 | | | 0,11 (0,08–0,15) | | 95,6% | 9 / 137.933 | 0,09 (0,06–0,15) | 98,5% | | | | | 3 / 1.694 | 0,0 (0,04–0,07) | | | 0,0% |
| 6 - 12 months | 13 / 3.791 | 0,20 (0,10–0,34) | 97,5% | 21 / 42.963 | | | 0,16 (0,10–0,25) | | 97,9% | 20 / 37.863 | 0,14 (0,08–0,24) | 96,8% | | | | | 10 / 3.760 | 0,13 (0,06–0,28) | | | 98,8% |
| > 12 months | 3 / 2.220 | 0,14 (0,09–0,22) | 89,3% | 4 / 848 | | | 0,24 (0,09–0,52) | | 94,6% | 4 / 2.247 | 0,23 (0,11–0,43) | 96,1% | | | | | 3 / 2.220 | 0,11 (0,06–0,19) | | | 93,3% |
| p – value between groups | (p = 0,5924) | | | (p = 0,1471) | | | | | | (p = 0,1314) | | | | | | | (p = 0,0188) | | | | |
| **Hospitalization** |  |  | | |  |  |  |  | | | | |  |  |  |  | | | | | |
| Yes | 14 / 6.388 | 0,15 (0,08–0,26) | 96,7% | 14 / 9.029 | | | 0,16 (0,10–0,26) | | 97,5% | 12 / 6.513 | 0,15 (0,07–0,29) | 96,8% | | | | | 10 / 4.850 | 0,09 (0,06–0,15) | | | 94,2% |
| No | - | - | - | 3 / 514 | | | 0,23 (0,06–0,57) | | 94,0% | 2 / 445 | 0,25 (0,03–0,76) | 93,4% | | | | | - | - | | | - |
| Some | 4 / 889 | 0,31 (0,19–0,46) | 92,3% | 13 / 41.720 | | | 0,14 (0,08–0,22) | | 97,7% | 12 / 36.774 | 0,15 (0,08–0,26) | 97,5% | | | | | 5 / 2.718 | 0,18 (0,05–0,50) | | | 99,4% |
| Absent information | 1/ 127.859 | 0,10 (0,10–0,11) | - | 5 / 6.278 | | | 0,12 (0,04–0,31) | | 95,2% | 7 / 134.311 | 0,08 (0,04–0,14) | 94,8% | | | | | 1 / 106 | 0,05 (0,02–0,11) | | | - |
| p – value between groups | (p = 0,0001) | | | (p = 0,8407) | | | | | | (p = 0,3256) | | | | | | | (p = 0,1902) | | | | |
| **Cohort** |  |  | | |  |  |  |  | | | | |  |  |  |  | | | | | |
| Retrospective | 5 / 129.821 | 0,08 (0,03–0,20) | 95,6% | 8 / 5.065 | | | 0,11 (0,07–0,17) | | 95,2% | 7 / 129.682 | 0,08 (0,03–0,17) | 98,1% | | | | | 5 / 3.743 | 0,05 (0,03–0,09) | | | 92,9% |
| Prospective | 14 / 5315 | 0,22 (0,14–0,34) | 97,4% | 27 / 52.476 | | | 0,17 (0,12–0,24) | | 97,7% | 26 / 48.361 | 0,16 (0,10–0,23) | 98,3% | | | | | 11 / 3.931 | 0,16 (0,08–0,28) | | | 98,6% |
| p – value between groups | (p = 0,0441) | | | (p = 0,1375) | | | | | | (p = 0,1149) | | | | | | | (p = 0,0090) | | | | |
| **Studies’ quality** |  |  | | |  |  |  |  | | | | |  |  |  |  | | | | | |
| Poor | - | - | - | 1 / 248 | | | 0,04 (0,02–0,07) | | - | 1 / 248 | 0,04 (0,02–0,07) | - | | | | | - | - | | | - |
| Fair | 19 / 135.136 | 0,17 (0,11–0,27) | 98,4% | 32 / 55.369 | | | 0,16 (0,12–0,22) | | 97,3% | 31 / 177.291 | 0,16 (0,10–0,21) | 99,7% | | | | | 16 / 7.674 | 0,11 (0,06–0,19) | | | 98,6% |
| Good | - | - | - | 2 / 1.924 | | | 0,10 (0,02–0,37) | | 98,2% | 1 / 504 | 0,03 (0,02–0,05) | - | | | | | - | - | | | - |
| p – value between groups | *-* | | | (p = 0,0001) | | | | | | (p < 0,0001) | | | | | | |  |  | - |  |  |
| **Study continent** |  |  | | |  |  |  |  | | | | |  |  |  |  | | | | | |
| Europe | 9 / 1.781 | 0,17 (0,08–0,34) | 95,2% | 19 / 47.079 | | | 0,15 (0,10–0,22) | | 97,5% | 19 / 43.352 | 0,12 (0,07–0,20) | 98,4% | | | | | 7 / 2.927 | 0,08 (0,04–0,16) | | | 94,0% |
| Asia | 2 / 1.767 | 0,11 (0,08–0,13) | 49,4% | 5 / 3.793 | | | 0,08 (0,05–0,12) | | 91,9% | 4 / 3.627 | 0,08 (0,03–0,19) | 92,6% | | | | | 1 / 534 | 0,12 (0,09–0,15) | | | - |
| North America | 3 / 128.442 | 0,28 (0,10–0,56) | 99,6% | 4 / 2.406 | | | 0,27 (0,08–0,61) | | 98,2% | 5 / 129.935 | 0,24 (0,08–0,52) | 99,5% | | | | | 3 / 1.049 | 0,26 (0,04–0,74) | | | 99,1% |
| South America | 5 / 3.146 | 0,16 (0,05–0,39) | 98,2% | 5 / 594 | | | 0,19 (0,06–0,44) | | 95,2% | 5 / 2.129 | 0,16 (0,07–0,32) | 97,0% | | | | | 5 / 3.164 | 0,09 (0,03–0,23) | | | 98,6% |
| Africa | - | - | - | 1 / 1.873 | | | 0,17 (0,16–0,19) | | - | - | - | - | | | | | - | - | | | - |
| Europe and South America | - | - | - | 1 / 1.796 | | | 0,16 (0,14–0,18) | | - | - | - | - | | | | | - | - | | | - |
| p – value between groups | (p = 0,1655) | | | (p = 0,0153) | | | | | | (p = 0,4166) | | | | | | | (p = 0,5357) | | | | |

**CI**: Confidence Interval; **I²**: Higgins' inconsistency index (heterogeneity). ᵃPop.: Number of studies and sample size in the subgroup
